# Prevalence of Preoperative Anxiety and Associated Factors in Hallux Valgus Surgery

**DOI:** 10.1002/jfa2.70138

**Published:** 2026-02-20

**Authors:** Pedro C. Ramírez‐Navarro, David Rodríguez‐Sanz, Jorge Velázquez‐Saornil, Alberto Sardón Iribarnegaray, Bernardino Basas‐García, Mario Suárez‐Ortiz, Santiago Nieto‐Farrán, Edurne Nieto‐Sanmartín

**Affiliations:** ^1^ International Doctoral School of the University of Almería Almería Spain; ^2^ Faculty of Podiatry Complutense University of Madrid Madrid Spain; ^3^ Faculty of Health Sciences Pontifical University of Salamanca Salamanca Spain; ^4^ Department of Orthopedic Surgery and Traumatology La Inmaculada Hospital Almería Spain; ^5^ Department of Podiatric Surgery Basas Clinical Center Salamanca Spain; ^6^ International Doctoral School of the University of Extremadura Badajoz Spain; ^7^ Department of Podiatric Surgery Nieto Farrán Clinical Center Vizcaya Spain

**Keywords:** APAIS, foot surgery, hallux valgus, minimally invasive surgery, preoperative anxiety

## Abstract

**Introduction:**

Preoperative anxiety is that which is motivated by the emotional and physical stressors inherent in a surgical intervention. The aim of this study was to assess preoperative anxiety as well as the associated sociodemographic and surgical factors in the context of a common foot surgery procedure, such as surgery for the correction of hallux valgus.

**Methods:**

A cross‐sectional study was conducted with a total of 80 patients using the Amsterdam Preoperative Anxiety and Information Scale (APAIS) during the preoperative consultation to determine the level of anxiety and information among patients undergoing osteoarticular surgery for the correction of hallux valgus. Additionally, a questionnaire was used to gather information on other variables such as age, gender, type of surgery, and surgical history.

**Results:**

36.3% of the participants experienced preoperative anxiety. Patients undergoing minimally invasive surgery had lower levels of preoperative anxiety compared to those undergoing conventional surgery (*p* = 0.006). 81.3% of the patients requested additional information about the surgical procedure, which was correlated with the level of anxiety with patients who requested additional information being 5.57 times more likely to experience preoperative anxiety (*p* = 0.034). There were no significant differences in anxiety levels between men and women.

**Conclusions:**

The prevalence of preoperative anxiety and the demand for information were high among the participants in this study. The patient’s demand for information is a predictive factor of preoperative anxiety. Other factors, such as knowing the surgeon or having undergone previous surgery, acted as protective factors against preoperative anxiety.

## 1 Introduction

Anxiety is considered one of the most common and necessary emotions in certain situations of our daily lives [1]. It often presents as an unpleasant sensation in which the individual experiences fear, distress, and/or worry in addition to symptoms of tension and metabolic alterations [2, 3].

This emotion often arises as a warning in the face of potential danger or a situation of uncertainty or stress with the aim of taking the necessary measures to address the possible threat. Therefore, in certain situations, it is considered necessary, as it improves performance and the ability to solve everyday problems [1, 2].

Preoperative anxiety is that which is triggered by the emotional and physical stressors inherent in a surgical procedure [4]. The most common physical symptoms of preoperative anxiety that patients present, and that healthcare professionals can perceive are: muscle tension, restlessness, shortness of breath, stuttering, difficulty expressing themselves, increased sweating, paleness among others [5, 6, 7].

The etiology of anxiety prior to a surgical procedure is varied; however, in most cases, it is triggered by the anesthetic process. This often generates insecurity in the patient primarily due to the fear that the local anesthetic may not work properly, cause pain, or produce an adverse effect on the body [8]. Another stressful factor for the patient is the potential consequences of surgical failure and what this could mean for their quality of life, such as recovery time, postoperative pain, physical disability, or even the risk of death [6, 9, 10].

There are other factors associated with anxiety prior to a surgical procedure, such as the patient’s surgical experience, the surgical technique to be performed, the type of anesthesia, the level of information provided to the patient as well as demographic and psychosocial factors [3].

Despite being an underrated aspect by surgeons [7, 11], it is important to understand the level of anxiety the patient is experiencing before the surgical procedure in order to improve their physical and mental state and to prevent potential complications prior to surgery by adapting to the needs of each individual case [8, 12]. Recent studies [13] assert that preoperative anxiety has unfavorable effects on the induction and maintenance of anesthesia. It also worsens pain perception and increases the risk of postoperative infections. Additionally, it is well known that high levels of preoperative anxiety can negatively interfere with the patient's postoperative recovery, increasing recovery time and postoperative pain levels [13, 14, 15, 16, 17, 18].

In recent decades, preoperative anxiety has been studied by numerous authors from various medical–surgical specialties [8, 12, 13]; however, there are no studies in the scientific literature addressing anxiety prior to surgical procedures in the field of minimally invasive osteoarticular foot surgery.

The present study aims to assess the level of preoperative anxiety and information using the Amsterdam Preoperative Anxiety and Information Scale [19, 20] in patients undergoing osteoarticular surgery for hallux valgus correction as well as to identify the sociodemographic and surgical factors associated with preoperative anxiety.

## 2 Materials and Methods

### 2.1 Settings

A cross‐sectional study was conducted from July 2023 to January 2024 registered in ClinicalTrials.gov with the code NCT05948748. The study has been approved by the AGSNA (Área de Gestión Sanitaria Norte de Almería) Health Care and Research Ethics Committee of the Hospital La Inmaculada de Almería (Spain) and that has been conducted in accordance with the principles set forth in the Helsinki Declaration. The participants were patients scheduled for hallux valgus surgery at “La Inmaculada” Hospital (Almería, Spain). All study participants signed a written informed consent document.

### 2.2 Sample Size Calculation

The theoretical universe of this study consists of patients who underwent surgery in previous years and who, based on the inclusion criteria, could be part of the study. It is estimated that around 8 surgeries are performed per month amounting to a total of 100 patients per year. Therefore, for the purposes of sample size calculation and sampling error, 100 cases are considered. According to previous studies [4], approximately 22.6% of patients may experience anxiety. Using GPower 3.1, it was determined that the sample size is 73 patients with a maximum allowable margin of error of 5% for a 95% confidence level considering a 10% loss rate. The minimum sample size calculated was approximately 73. However, 80 cases were recorded for our study.

### 2.3 Participants

The participants in this study were patients who underwent surgery to correct hallux valgus deformity. The surgical procedure involved the correction of the hallux valgus deformity under local anesthesia using the appropriate technique depending on the degree of deformity and the surgeon’s discretion. The inclusion criteria were: being over 16 years old, patients classified as ASA I and II according to the American Society of Anesthesiologists (ASA) and having the autonomy to participate and complete the study forms. The exclusion criteria were: patients with emotional, psychological, or psychiatric disorders, those unable to understand or respond to the study questions, those undergoing treatment with central nervous system depressants, or those with severe toxic habits.

### 2.4 Data Collection

Data collection was carried out during the preoperative consultation, before administering local anesthesia to the patient with the aim of recording the moment of highest preoperative anxiety, as indicated by other authors [4, 6, 21]. The Amsterdam Preoperative Anxiety and Information Scale (APAIS) was used, as it is a specific scale for evaluating preoperative anxiety and the demand for information about the surgery (additional information beyond the legal explanation of the procedure) and is validated in Spain [20]. As in other studies [4, 20], before the surgery, a questionnaire was also provided to gather information on sociodemographic and surgical factors related to preoperative anxiety, such as: gender, age, body mass index (BMI), education level, surgical and anesthetic history, and whether they knew the surgeon.

The APAIS scale was designed by Moerman [19] in 1996. The survey consists of six questions to which the patient must respond using a 5‐point Likert scale where 1 = not at all, 2 = very little, 3 = somewhat, 4 = quite a bit, and 5 = a lot. The APAIS scale consists of 6 items: (1) “I am worried about the anesthesia,” (2) “I constantly think about the anesthesia,” (3) “I would like to know as much as possible about the anesthesia,” (4) “I am worried about the operation,” (5) “I constantly think about the operation,” (6) “I would like to know as much as possible about the operation.”

On one hand, the first and second questions are related to anxiety about the anesthesia procedure, whereas the fourth and fifth questions evaluate anxiety regarding the surgical procedure. The anxiety score is obtained by adding the scores assigned to items 1, 2, 4, and 5. After adding the scores, the result will range between 4 and 20 points with patients considered anxious, according to the original version of the scale, if their results are ≥ 11 points [19].

On the other hand, questions three and six refer to the patient’s demand for information about the anesthesia and surgery. The sum of items 3 and 6 was calculated to assess the need for more information. After summing the subject’s score, the results will range between 2 and 10 points with patients scoring ≥ 5 being those who required additional information about the anesthesia or surgery according to the original version of the scale [19].

### 2.5 Statistical Analysis

For the descriptive statistical analysis of the sample, the number of cases in each category and the corresponding percentage were obtained for qualitative variables. For quantitative variables, the minimum, maximum, mean, and standard deviation (SD) values were calculated.

The comparison between groups for qualitative variables was performed using the Chi‐square test. For the comparison of means between two groups, the t‐Student test was used after checking for normality (Kolmogorov–Smirnov test) and homogeneity (Levene’s test). The forward stepwise Wald multivariate logistic regression model was used to determine the effect of demographic and surgical variables in relation to preoperative anxiety and the demand for information.

The statistical analysis was performed using SPSS 25.0 for Windows. Differences were considered statistically significant if *p* < 0.05.

## 3 Results

**FIGURE 1:**
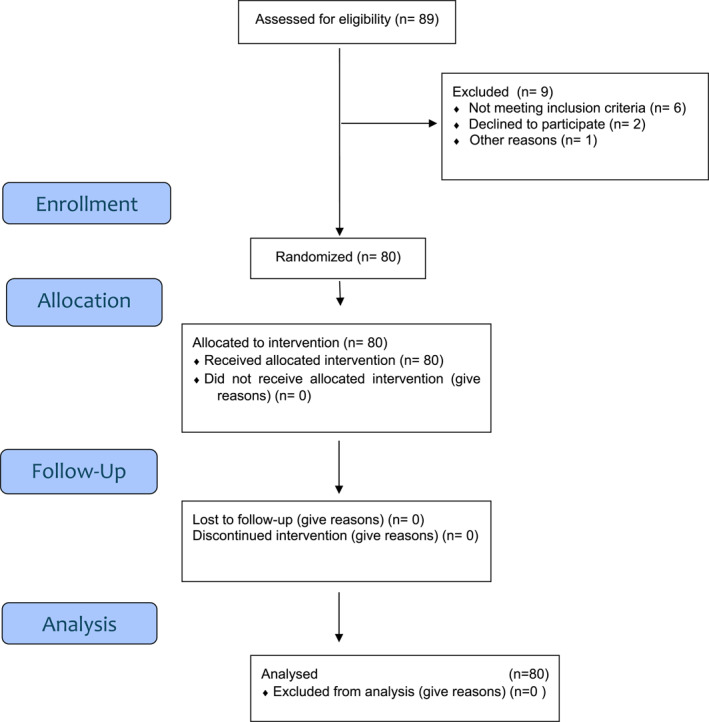
Flow diagram.

The final sample of the study consisted of 80 patients (Figure 1) of whom 11.3% (*n* = 9) were men and 88.8% (*n* = 71) were women with ages ranging from 16 to 89 years and an average age of 62.4 years (SD = 13.9). According to the classification of patients based on the scores on the APAIS scale (Table 1), 63.7% did not show anxiety, and 18.8% did not request additional information about the surgery.

**TABLE 1:** Descriptive statistics of patients based on APAIS scale.

	*N*	%
Anxiety		
No (< 11)	51	63.7
Yes (≥ 11)	29	36.3
Information		
No (< 5)	15	18.8
Yes (≥ 5)	65	81.3

Below are the results of the logistic regression models performed at both the univariate and multivariate levels to determine the demographic and surgery‐related variables that have a statistically significant effect on anxiety and the need for information.

In relation to anxiety, at the univariate level (Table 2), the variables that showed statistically significant effects were: the need for information (patients who requested more information about the surgery were 4.62 times more likely to have anxiety than those who did not request it); and the variables of the type of surgery, having undergone surgery before and knowing the surgeon, all of which acted as protective factors against anxiety.

**TABLE 2:** Descriptive and univariate logistic regression on anxiety.

	Anxiety		Univariate logistic regression	
	No (< 11)	Yes (≥ 11)	OR (CI 95%)	*p* value
Age, media (DT)	63.71 (12.64)	60.1 (15.78)	0.98 (0.95–1.01)	0.266
BMI, *n* (%)				
Normal weight	15 (29.4)	10 (34.5)	1	
Overweight	26 (51)	16 (55.2)	0.92 (0.34–2.54)	0.877
Obesity	10 (19.6)	3 (10.3)	0.45 (0.10–2.05)	0.303
Education level, *n* (%)				
University studies	17 (33.3)	10 (34.5)	1	
Non university studies	34 (66.7)	19 (65.5)	0.95 (0.36–2.49)	0.917
APAIS information, *n* (%)				
< 5	13 (25.5)	2 (6.9)	1	
≥ 5	38 (74.5)	27 (93.1)	4.62 (1.16–22.16)	**0.044**
Type of surgery, *n* (%)				
Others	5 (9.8)	8 (27.6)	1	
Minimally invasive	46 (90.2)	21 (72.4)	0.29 (0.08–0.98)	**0.046**
Type of anesthesia, *n* (%)				
With sedation	19 (37.3)	12 (41.4)	1	
Without sedation	32 (62.7)	17 (58.6)	0.84 (0.33–2.14)	0.716
Operated before, *n* (%)				
No	13 (25.5)	15 (51.7)	1	
Yes	38 (74.5)	14 (48.3)	0.32 (0.12–0.84)	**0.020**
Knowing the surgeon, *n* (%)				
No	17 (33.3)	19 (65.5)	1	
Yes	34 (66.7)	10 (34.5)	0.26 (0.10–0.69)	**0.007**

*Note:* Numbers in bold indicate that they are statistically significant.

Abbreviations: CI, confidence interval; OR, odds ratio.

At the multivariate level (Table 3), the variables continue to show a significant effect: the need for information (patients who requested more information about the surgery were 5.57 times more likely to have anxiety than those who did not request it) as well as the variables type of surgery and knowing the surgeon, both of which acted as protective factors, reducing the likelihood of experiencing anxiety.

**TABLE 3:** Multivariate logistic regression model for anxiety.

	B (ET)	Wald	OR (CI 95%)	*p* value
Age	−0.02 (0.02)	0.47	0.99 (0.94–1.03)	0.495
BMI				
Normal weight				
Overweight	0.00 (0.62)	0.00	1.00 (0.30–3.37)	0.997
Obesity	−0.51 (0.91)	0.31	0.60 (0.10–3.57)	0.575
University studies (yes vs. no)	0.59 (0.70)	0.70	1.80 (0.46–7.08)	0.403
APAIS information (yes vs. no)	1.72 (0.81)	4.50	5.57 (1.14–27.18)	**0.034**
Minimally invasive surgery (yes vs. no)	−1.48 (0.54)	7.43	0.23 (0.08–0.66)	**0.006**
Type of anesthesia (with vs. without sedation)	−0.72 (0.59)	1.49	0.49 (0.15–1.55)	0.223
Operated before (yes vs. no)	−0.66 (0.62)	1.14	0.52 (0.15–1.74)	0.285
Knowing the surgeon (yes vs. no)	−1.34 (0.61)	4.90	0.26 (0.08–0.86)	**0.027**

*Note:* Numbers in bold indicate that they are statistically significant.

Abbreviations: CI, confidence interval; OR, odds ratio.

For the request for information, at the univariate level (Table 4), the variables that showed statistically significant effects were: education (patients without university education are 5.26 (1/0.19) times more likely not to request information than those with university education) and the variable knowing the surgeon (patients who know the surgeon are 3.7 (1/0.27) times more likely not to request information than those who do not know the surgeon).

**TABLE 4:** Descriptive univariate logistic regression on anxiety.

	Information		Regresión logística univariante	
	No (< 5)	Yes (≥ 5)	OR (CI 95%)	*p* value
Age, media (DT)	66.8 (11.85)	61.38 (14.18)	0.97 (0.92–1.02)	0.175
BMI, *n* (%)				
Normal weight	3 (12)	22 (88)		
Overweight	10 (23.8)	32 (76.2)	0.44 (0.11–1.77)	0.246
Obesity	2 (15.4)	11 (84.6)	0.75 (0.11–5.17)	0.770
Education level, *n* (%)				
University studies	2 (44.4)	25 (55.6)		
Non university studies	18 (20.8)	35 (79.2)	0.19 (0.05–0.77)	**0.007**
Type of surgery, *n* (%)				
Others	1 (7.7)	12 (92.3)		
Minimally invasive	14 (20.9)	53 (79.1)	0.32 (0.04–2.64)	0.287
Type of anesthesia, *n* (%)				
With sedation	8 (25.8)	23 (74.2)		
Without sedation	7 (14.3)	42 (85.7)	2.09 (0.67–6.49)	0.204
Operated before, *n* (%)				
No	4 (14.3)	24 (85.7)		
Yes	11 (21.2)	41 (78.8)	0.27 (0.09–0.85)	**0.019**
Knowing the surgeon, *n* (%)				
No	7 (19.4)	29 (80.6)		
Yes	8 (18.2)	36 (81.8)	1.09 (0.35–3.35)	0.886

*Note:* Numbers in bold indicate that they are statistically significant.

Abbreviations: CI, confidence interval; OR, odds ratio.

At the multivariate level (Table 5), the variables continue to show a significant effect: education (patients without university education are 5.26 (1/0.19) times more likely not to request information than those with university education) and knowing the surgeon (patients who know the surgeon are 3.7 (1/0.27) times more likely not to request information than those who do not know the surgeon).

**TABLE 5:** Multivariate logistic regression model for information.

	B (ET)	Wald	OR (CI 95%)	*p* value
Age	−0.02 (0.03)	0.45	0.98 (0.93–1.04)	0.504
BMI				
Normal weight	1			
Overweight	−0.86 (0.77)	1.24	0.42 (0.09–1.93)	0.266
Obesity	−0.31 (1.05)	0.08	0.74 (0.09–5.77)	0.772
University studies (yes vs. no)	1.46 (0.64)	5.23	4.29 (1.23–14.96)	**0.022**
Minimally invasive surgery (yes vs. no)	−0.53 (1.22)	0.19	0.59 (0.05–6.42)	0.666
Type of anesthesia (with vs. without sedation)	1.02 (0.65)	2.45	2.77 (0.77–9.91)	0.117
Operated before (yes vs. no)	−0.63 (0.30)	4.56	0.53 (0.30–0.95)	**0.033**
Knowing the surgeon (yes vs. no)	0.07 (0.70)	0.01	1.07 (0.27–4.21)	0.923

*Note:* Numbers in bold indicate that they are statistically significant.

Abbreviations: CI, confidence interval; OR, odds ratio.

## 4 Discussion

The present study aimed to assess preoperative anxiety and its associated factors in relation to a common surgical procedure in foot surgery, namely surgery for the correction of hallux valgus. As in other studies with an acceptable level of reliability [6, 13, 21, 22, 23], the Amsterdam Preoperative Anxiety and Information Scale (APAIS) was used to determine the patient’s level of preoperative anxiety, as it is a specific tool for evaluating preoperative anxiety and the demand for additional information about the surgery, which is also validated in Spain [20].

The main finding of the present study showed that preoperative anxiety in minor outpatient foot surgery for the correction of hallux valgus was present in 36.3% (29 out of 80 participants) according to the results of the APAIS scale. These results were similar to those obtained by other authors who used the same scale in different types of surgeries. For example, in the study by Gu et al., they found that 30% of patients undergoing laparoscopic gynecological surgery experienced anxiety [21], Suman Prasad Adhikari reported 31% anxiety before gastrointestinal surgeries [13], and Eberhart et al. recorded 40.5% preoperative anxiety in patients undergoing elective surgery [6]. Other studies reported significantly higher levels of preoperative anxiety ranging from 47% to 82.1% [24, 25, 26, 27, 28], which could be related to the region where the study was conducted, as most of these studies were carried out in Africa. Additionally, factors such as sample size, type of surgical procedure, and anesthetic method could explain these higher percentages of preoperative anxiety.

In line with other studies [4, 13], no significant differences in preoperative anxiety were found between men and women. Other authors [7, 9, 29, 30, 31] report that preoperative anxiety was higher in women than in men, which can be attributed to the fact that, generally, women tend to express their feelings more easily. Additionally, the patient’s age was not an influential factor in preoperative anxiety, as reflected in the study by Yilmaz et al. [32] on preoperative anxiety in elective surgery.

Regarding the surgical factors studied in the present work, it was observed that patients undergoing minimally invasive surgery showed lower levels of preoperative anxiety compared to those undergoing conventional surgery (*p* = 0.006). This could be due to the fact that minimally invasive surgery is performed through smaller incisions, which reduce postoperative recovery time, making it a less aggressive surgical procedure. Additionally, it offers good results in patients with mild to moderate hallux valgus and has a lower likelihood of postoperative complications [33, 34].

Patients with prior surgical and anesthetic experiences had lower levels of anxiety compared to those without such experiences (*p* = 0.020) similar to data reported by other authors [9, 13]. Therefore, having undergone surgery could reduce the likelihood of experiencing preoperative anxiety. Additionally, there was a statistically significant difference between patients who did not know the surgeon and those who did with those who knew the surgeon showing lower levels of preoperative anxiety (*p* = 0.007). Thus, knowing the person who will perform the surgery could act as a protective factor against preoperative anxiety. Kocaturk et al. [35] stated in their study on preoperative anxiety in oral and maxillofacial surgery that a prior in‐person interview, allowing the patient to meet the surgeon who will operate on them, significantly reduces preoperative anxiety levels and decreases the likelihood of postoperative complications.

On the other hand, regarding the demand for additional information beyond the legal explanation of the procedure, and according to the classification of patients based on the information demand scores from the APAIS scale, 81.3% of the patients requested additional information about the surgical procedure (65 out of 80 participants). In the recent study by Adhikari et al. [13] on preoperative anxiety in elective surgery, 64.9% of the population (133 out of 205 participants) requested additional information about the surgical procedure in gastrointestinal, hepatobiliary, urological, and orthopedic surgeries, a result similar to ours. However, Navarro’s study [4] on preoperative anxiety in foot surgery found that 43.9% of patients needed additional information about the surgical procedure. This lower result compared to our study may be due to the type of surgery performed, as in our case, patients underwent osteoarticular surgery for hallux valgus correction, whereas in the previously cited study, a soft tissue procedure (nail surgery) was performed. This type of surgery might generate less uncertainty and demand for information from patients, as it is a simpler procedure with generally lighter postoperative care. This leads us to believe that it might be beneficial to provide additional information about the surgical procedure to patients undergoing osteoarticular foot surgery in order to meet their information needs and reduce the likelihood of experiencing preoperative anxiety.

Regarding the demand for information, it was observed that patients without university education were 5.26 times more likely not to request additional information about the surgical procedure (*p* = 0.007). This could be attributed to the fact that a lower educational level may lead to less interest in the procedure and potential postoperative complications. However, the study by Gürler et al. [29] concluded that participants with no education beyond primary school showed higher levels of preoperative anxiety. Therefore, the patient's educational level may be a factor to consider when communicating information about the surgical procedure.

Regarding the fact of knowing the surgeon who was going to perform the procedure, it was observed that patients who knew the surgeon were 3.7 times more likely not to request additional information than those who did not know the surgeon. This can be attributed to the fact that knowing the surgeon and having had the opportunity to meet personally with them generates greater confidence in the patient, thereby also reducing the likelihood of experiencing preoperative anxiety. According to the scientific literature [9, 35], presurgery consultations with the surgeon and/or anesthesiologist significantly reduce the probability of preoperative anxiety.

The demand for additional information about the surgical procedure (APAIS ≥ 5) was related to preoperative anxiety (APAIS ≥ 11) with patients who requested information being 5.57 times more likely to experience anxiety than those who did not request it (*p* = 0.034). Thus, the demand for information could be a good predictor of preoperative anxiety. These results align with those of Martel et al. [36], who found a correlation between the demand for information and higher levels of preoperative anxiety in patients undergoing intravitreal injections. Other authors [37] concluded that the need for information in patients undergoing elective craniotomy for supratentorial neoplasms is a predictor of preoperative anxiety. Atar et al. [38] pointed out that the lack of information among patients undergoing nasal surgery is also a significant source of stress, and they propose that preoperative information should be provided to the patient before scheduling the surgery. Other recent studies [35, 39, 40, 41] emphasize the importance of educational and informational interviews about the planned surgical procedure, as they effectively reduce preoperative anxiety levels and decrease the likelihood of postoperative complications.

The present study has certain limitations. On one hand, the majority of the study population consisted of women, as they more frequently present deviations and alterations in the toes, such as hallux valgus. This prevented us from comparing preoperative anxiety equally between gender groups. On the other hand, since this was a multicenter study, the surgical and anesthetic procedures were performed by a total of four surgeons, meaning that each patient was treated by a different team, which could result in differences in patient care and the sense of trust conveyed to them. Consequently, this may lead to variations in the patient’s anxiety level. However, this could also be considered a strength, as it provides a more comprehensive view of preoperative anxiety in foot surgery. Another strength we highlight is the use of the APAIS scale, as it is a specific tool for evaluating preoperative anxiety and the demand for information about the surgery, and it is validated in Spain.

Preoperative anxiety in the field of foot surgery is a relatively under‐researched area. Further studies are needed to gain a deeper understanding of the prevalence of preoperative anxiety in this part of the body as well as the factors that may influence it.

## 5 Conclusions

In conclusion, both preoperative anxiety and the demand for information were notably present in the subjects who participated in our study. Anxiety levels were lower in patients who underwent minimally invasive surgery. Other factors, such as knowing the surgeon or having undergone previous surgeries, acted as protective factors against preoperative anxiety. The patient’s demand for information is a predictor of preoperative anxiety. We consider it important to assess preoperative anxiety levels in foot surgery in order to reduce them and thus improve the surgical process and the patient's experience.

## Author Contributions

**Pedro C. Ramírez Navarro:** conceptualization, methodology, investigation, writing original draft preparation, writing – review and editing, project administration. **David Rodríguez‐Sanz:** conceptualization, methodology, formal analysis. **Jorge Velázquez‐Saornil:** validation, formal analysis, visualization. **Alberto Sardón Iribarnegaray:** investigation, resources. **Bernardino Basas‐García:** investigation, resources. **Mario Suárez‐Ortiz:** investigation, resources. **Santiago Nieto‐Farrán:** funding acquisition, writing – review and editing. **Edurne Nieto‐Sanmartín:** visualization, supervision.

## Funding

The authors have nothing to report.

## Ethics Statement

The study has been approved by the AGSNA (Área de Gestión Sanitaria Norte de Almería) Health Care and Research Ethics Committee of the Hospital La Inmaculada de Almería (Spain).

## Consent

Patients gave their informed consent to participate in the study and also confirmed their consent to the publication of data.

## Conflicts of Interest

The authors declare no conflicts of interest.

## Data Availability

Data are available on request from the corresponding author.
